# Antipsychotic Optimisation on an Adult Acute Inpatient Ward: A Retrospective Audit

**DOI:** 10.1192/bjo.2022.442

**Published:** 2022-06-20

**Authors:** Soracha Healy, Radhika Lakhani

**Affiliations:** South West London and St George's NHS Mental Health Trust, London, United Kingdom

## Abstract

**Aims:**

Antipsychotic prescribing in acute inpatient settings is an integral part of patient care. The aim of this audit was to review optimisation of antipsychotics on an acute adult inpatient ward in South West London and St George's NHS Mental Health Trust (SWLSTG). It was ascertained how antipsychotic prescribing on an acute ward meets NICE standards, including duration of antipsychotic use prior to medication change. Furthermore, communication of medication changes was reviewed in the context of the paramount importance of collaborative decision-making in aiding adherence. NICE recommends a 4–6 week trial of antipsychotic medication at optimal dosage. However, it was hypothesised this may vary with side-effects, adherence and risk management in the inpatient environment. To establish the relationship between these factors, data were extracted regarding antipsychotic counselling, baseline physical health investigations, antipsychotic choice, dosage and duration, side-effects and treatment response.

**Methods:**

Retrospective data analysis was conducted for patients on an acute adult inpatient ward in SWLSTG. Data extracted from Rio clinical record system and EPMA prescribing software were analysed in Excel. The inclusion criteria were patients admitted or transferred to a 20-bed acute mixed adult ward from 04/08/21 to 04/11/21 with a diagnosis of schizoaffective disorder, schizophrenia, bipolar affective disorder or nonorganic psychosis. This included patients being initiated or continued on antipsychotic medication. From 71 patients, 33 met inclusion criteria. Data were extracted regarding duration of treatment prior to changes in treatment, counselling and pre-treatment investigations. Furthermore, simple statistical analyses were carried out.

**Results:**

The most commonly initiated antipsychotics on admission were olanzapine (33%), quetiapine (21%), risperidone (15%) and zuclopenthixol decanoate (15%). In those requiring change in antipsychotic regime, mean duration from the start of treatment as inpatient to first change was 11.6 days, time between first and second change 13.8 days, and between second and third change 16.0 days.

The data showed in the majority (84.6%) antipsychotics were changed or up-titrated due to inadequate response. Out of the remainder 9.6% were changed due to intolerable side effects and 5.8% due to adherence concerns. In 73% of cases counselling was attempted regarding initial medication changes.

**Conclusion:**

Antipsychotic therapy was altered more quickly than advised by NICE guidance in the acute inpatient setting evaluated. This can be explained by increased risk, need for intensive management and individual clinical factors including side effects and adherence. Collaborative decision making could be enhanced by ensuring that counselling is attempted for every patient.

